# Dpp regulates autophagy-dependent midgut removal and signals to block ecdysone production

**DOI:** 10.1038/s41418-018-0154-z

**Published:** 2018-06-29

**Authors:** Donna Denton, Tianqi Xu, Sonia Dayan, Shannon Nicolson, Sharad Kumar

**Affiliations:** Centre for Cancer Biology, University of South Australia and SA Pathology, GPO Box 2471, Adelaide, SA 5001 Australia

**Keywords:** Autophagy, Development

## Abstract

Animal development and homeostasis require the programmed removal of cells. Autophagy-dependent cell deletion is a unique form of cell death often involved in bulk degradation of tissues. In *Drosophila* the steroid hormone ecdysone controls developmental transitions and triggers the autophagy-dependent removal of the obsolete larval midgut. The production of ecdysone is exquisitely coordinated with signals from numerous organ systems to mediate the correct timing of such developmental programs. Here we report an unexpected role for the *Drosophila* bone morphogenetic protein/transforming growth factor β ligand, Decapentaplegic (Dpp), in the regulation of ecdysone-mediated midgut degradation. We show that blocking Dpp signaling induces premature autophagy, rapid cell death, and midgut degradation, whereas sustained Dpp signaling inhibits autophagy induction. Furthermore, Dpp signaling in the midgut prevents the expression of ecdysone responsive genes and impairs ecdysone production in the prothoracic gland. We propose that Dpp has dual roles: one within the midgut to prevent improper tissue degradation, and one in interorgan communication to coordinate ecdysone biosynthesis and developmental timing.

## Introduction

In animal development tissue and organ morphogenesis is dependent on an intricate balance between cell division and programmed cell death (PCD) [1]. Although caspase-dependent apoptosis is the primary mode of developmental PCD, context specific modes of cell death that are dependent on autophagy, the catabolic process of cellular self-digestion through the action of lysosomal enzymes, also play a critical role [2–5]. Steroid hormones are important regulators of physiology and developmental transitions such as puberty in mammals and metamorphosis in insects [6]. The hormone-mediated juvenile-to-adult transition requires the remodeling or removal of juvenile structures by PCD. In *Drosophila*, the major developmental transitions are triggered by the steroid hormone 20-hydroxyecdysone (20E, ecdysone). The hormone binds its heterodimeric receptor, ecdysone receptor/ultraspiracle (EcR/Usp), in a spatiotemporal manner to regulate proliferation, differentiation, and PCD [7–10]. Multiple environmental and developmental signals coordinate hormone production to regulate developmental transitions and overall body size. However, the molecular links integrating hormone biosynthesis with other signals require further investigation.

A large ecdysone pulse just prior to the larval–pupal transition triggers metamorphosis accompanied by the degradation of the larval midgut characterized by the initial contraction of the gastric caeca which requires an autophagy-dependent cell death mechanism [11]. In addition to the hormonal cues, down-regulation of growth signaling through Ras and PI3K is critical for the appropriate induction of autophagy-dependent midgut PCD [12]. Here, we identify the bone morphogenetic protein/transforming growth factor β (BMP/TGF-β) ligand, decapentaplegic (Dpp), a morphogen required for developmental patterning [13], as an essential regulator of autophagy-dependent midgut degradation. The canonical Dpp signaling pathway in *Drosophila* is similar to that in mammals [14]. Signaling is initiated by Dpp binding to a type II receptor (Punt/Put), which recruits and phosphorylates a type I receptor (Thickveins/Tkv or Saxophone/Sax). The type I receptor phosphorylates and activates the Receptor-Smad (R-Smad) Mothers against dpp (Mad), which then binds to the common-Smad (co-Smad) Medea (Med). The active R-Smad/co-Smad (Mad-Med) complex translocates to the nucleus to regulate target gene expression. Inhibitory Smads (Daughters against Dpp/Dad) act as a negative feedback control by competing with R-Smads [14]. Several studies have implicated TGF-β in the regulation of autophagy [15–17]. In certain cancer cell lines, TGF-β has been shown to stimulate autophagy, with upregulation of autophagy genes *Beclin-1*, *Atg5*, and *Atg7* [15, 17]. Similarly, TGF-β1 treatment of kidney cells resulted in increased reactive oxygen species and autophagy [18]. Sustained TGF-β can induce either autophagy or apoptosis depending on context [16], and TGF-β1 has also been implicated in down-regulating excessive autophagy [19]. Thus the physiological relationship between TGF-β signaling and autophagy-dependent cell death remains poorly understood. In this study we describe an unexpected and novel role of Dpp signaling in larval midgut growth and inhibition of autophagy. We found that an interaction between Dpp and ecdysone signaling plays a crucial function in regulating autophagy-dependent midgut degradation.

## Results

### Dpp prevents the premature removal of larval midgut cells

**Fig. 1:**
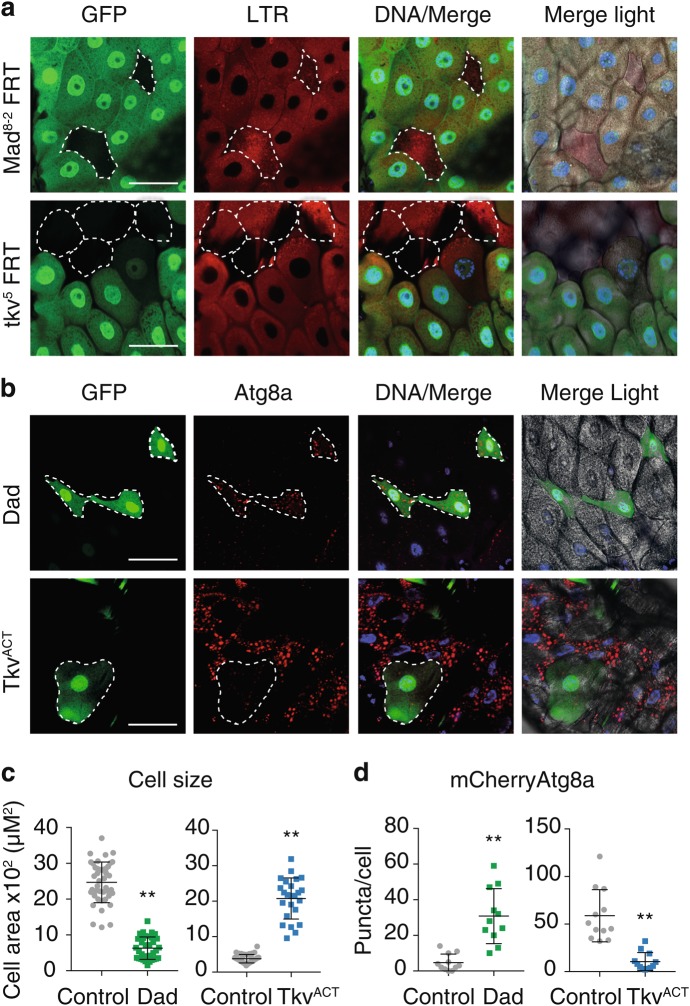
Dpp signaling prevents cell size contraction and autophagy during larval midgut degradation. **a** Loss-of-function clones of *Mad* and *tkv* die prematurely. *Mad*^*8-2*^ *(hsFLP; Mad*^*8-2*^ *FRT-40A/Ubi-GFP FRT-40A)* or *tkv*^*5*^ *(hsFLP tkv*^*5*^ *FRT-40A/Ubi-GFP FRT-40A)* homozygous cells detected by the absence of GFP are rapidly removed compared to the neighboring control cells marked by GFP (green) at −4 h RPF. LysoTracker red (red) and DNA stained by Hoechst (blue). Scale bar represents 25 μm. Live imaging reveals the rapid degradation of the *Mad*^*8-2*^ and *tkv*^*5*^ mutant cells (outlined), and show different stages of degradation. Due to the rapid degradation of the mutant cells, they are difficult to image. **b** Blocking Dpp signaling induces premature autophagy with small cell size while activating Dpp signaling reduces autophagy with larger cell size. Dad-expressing cells *(hsFLP; pmCherry-Atg8a/UAS-Dad; Act* *>* *CD2* *>* *GAL4, UAS-nlsGFP/+)* marked by GFP (green) have increased levels of mCherry-Atg8a puncta (red, outlined) compared to the neighboring control cells and smaller cell size at −8 h RPF. Tkv^ACT^-expressing cell *(hsFLP; pmCherry-Atg8a/+; Act* *>* *CD2* *>* *GAL4, UAS-nlsGFP/UAS-tkv*^*ACT*^*)* marked by GFP (green) has decreased levels of mCherry-Atg8a puncta (red, outlined) compared to the neighboring control cells and larger cell size from +1 h RPF gastric caeca. DNA is stained by Hoechst (blue). Scale bar represents 25 μm. **c** Quantitation of cell size from (**b**) measured using ImageJ (average ± SD) (^**^p < 0.0001, compared to control). **d** Quantitation of puncta per cell from (**b**) measured using ImageJ (average ± SD.) (^**^p *<* 0.0001, compared to control)

The ecdysone-triggered larval gut removal requires autophagy and cell size reduction. To identify regulators of this process we investigated clonal cells with loss of Dpp signaling and found that clones of *Mad* or *tkv* mutant cells underwent rapid elimination just prior to the initiation of the programmed larval midgut removal. Intact clones homozygous for *Mad*^8–2^ were difficult to find and image as the mutant cells in the midgut appeared to be rapidly removed, often showing degraded subcellular contents (Fig. 1a). This dramatic cell death was also observed in cell clones homozygous for *tkv* [5] (Fig. 1a), thus demonstrating an essential role for Dpp signaling in survival of midgut cells. Examination of autophagy using mChery-Atg8a revealed large puncta present in the rapidly dying cells, with abnormally diffuse DNA (Supplementary information, Figure S1a). The surrounding wild-type cells that have also initiated autophagy-dependent PCD remain intact as expected at this stage of PCD. This unique morphology of dying *Mad* and *tkv* deficient cells suggests that autophagy-dependent cell death may occur by the bulk degradation of cellular components. In addition, feeding the larvae chloroquine resulted in a partial rescue of the rapid removal of these cells observed by the presence of intact nuclei (Supplementary information, Figure S1b). Chloroquine inhibits lysosomal enzyme function and prevents autophagosomal fusion and degradation, highlighting the important role of autophagy in the removal of the midgut cells.

To further characterize the role of Dpp in autophagy-dependent cell death we generated clones in the midgut that give rise to cells-expressing Dad (an inhibitor of Dpp signaling) adjacent to control cells. The Dad clone cells had a less severe phenotype compared to the *Mad* or tk*v* mutant cells, enabling a more detailed analysis of the timing of autophagy induction and cell size contraction. The Dad-expressing cells were smaller in size with increased levels of autophagy at an early stage (−8 h RPF) compared to the neighboring control cells indicating the premature onset of autophagy-dependent cell removal (Fig. 1b–d). Conversely, to determine the effect of sustained Dpp signaling we examined the expression of the constitutively active mutant form of the receptor Thickveins, Tkv^Q253D^ (referred to as Tkv^ACT^) [20]. Clone cells-expressing Tkv^ACT^ in the midgut were larger with reduced autophagy compared to the neighboring control cells following the onset of midgut PCD (+1 h RPF) indicating a delay in their removal (Fig. 1b–d). At an earlier stage (−8 h RPF) prior to the initiation of PCD the Tkv^ACT^ cells were similar in size to the adjacent wild-type cells indicating that Tkv^ACT^ does not influence midgut cell size in third instar larvae, but rather prevents the cell size contraction during cell death (Supplementary information, Figure S1c). These findings reveal that Dpp signaling blocks autophagy-dependent PCD in vivo, and that down-regulation of Dpp signaling is required for the correct timing of midgut removal.

### Dpp signaling in the larval midgut blocks degradation

**Fig. 2:**
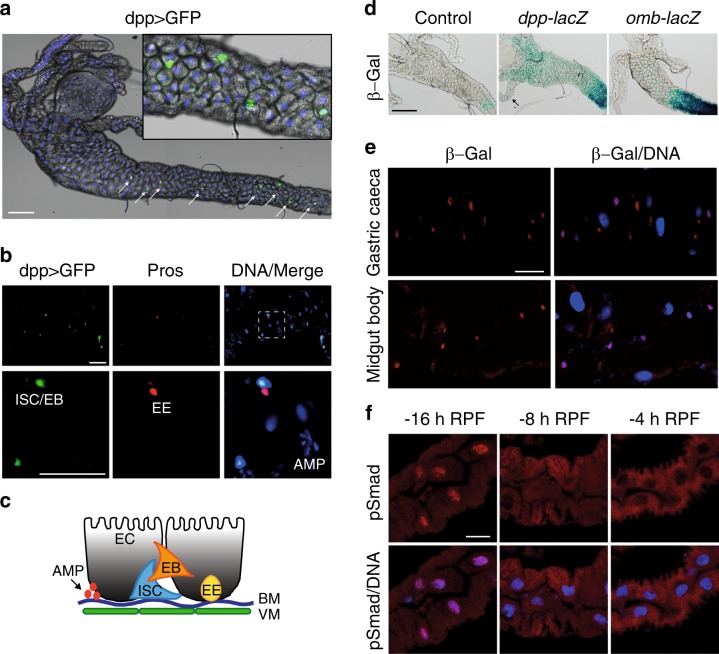
Dpp expression and activity in the midgut. **a** Dpp-GAL4 driving UAS-GFP as a marker for Dpp expressing midgut cells (Dpp > GFP). GFP-positive cells (green) can be found along the anterior midgut (arrows). Inset shows higher magnification. Scale bar represents 100 μm. **b** Dpp > GFP (green) is expressed in the larval intestinal stem cells (ISC)/enteroblasts (EB) and not in the Prospero-labeled enteroendocrine (EE) cells. Higher magnification inset shows the GFP and Pros cells do not colocalize. DNA is stained by Hoechst (blue). Scale bar represents 50 μm. **c** Schematic representation of larval midgut cells. The larval midgut cells include intestinal stem cells (ISC), enteroblasts (EB), enteroendocrine (EE), and enterocytes (EC) cells. Other cell types include adult midgut progenitors (AMP), and the basement membrane (BM) and visceral muscle (VM). **d** In control, *dpp-lacZ* and *omb-LacZ* expression of LacZ reporter for Dpp signaling can be detected by β-GAL activity in the midgut at −4 h RPF. X-gal activity is present in distinct cells along the gastric caeca (arrow) and along the midgut. Scale bar represents 100 μm. **e** β-GAL immunolocalization in *dpp-LacZ* midguts at −4 h RPF marks distinct cells along the gastric caeca and along the midgut body. Scale bar represents 20 μm. **f** Nuclear localization of phospho-Smad (red) and DNA (blue) in midgut ECs is high in −16 h RFP, with reduced levels in −8 h RPF and low-nuclear levels in −4 h RPF. Scale bar represents 10 μm

Although Dpp activity is known to be present in the larval midgut [21–24], the function of Dpp in this tissue remains unknown. To examine the spatial localization of Dpp we used dpp-GAL4 to drive expression of UAS-GFP (dpp > GFP), which revealed distinct GFP-positive cells in the midgut (Figs. 2a, b). The larval midgut comprises a number of cell types including the intestinal stem cells (ISCs), which give rise to a new ISC and an enteroblast (EB) that differentiates into the absorptive enterocyte (EC) or enteroendocrine cell (EE) (Fig. 2c). The dpp > GFP (green) was detected in distinct GFP-positive ISC and EB, but EE cells that are identified by Prospero expression lacked GFP (Fig. 2b). Based on nuclear size and location, GFP was not detected in the ECs or the undifferentiated adult midgut progenitors identified as clusters of small cells [25]. Additionally, using reporters for Dpp activity, *dpp*-*lacZ* [26] and *omb-lacZ*, β-GAL activity could be detected along the midgut, consistently expressed at high levels in the middle part of the anterior midgut (Figure 2d). β-GAL immunostaining of *dpp*-lacZ confirmed this localization and identified specific cells in the midgut with strong staining consistent with the dpp > GFP cell localization (Fig. 2e). Nuclear phospho-Smad (p-Smad) staining, a marker of Dpp signaling activity, was detected in feeding third instar larval midguts (−16 h RPF) that decreased in wandering (−8 h RPF), and late third instar larval midguts (−4 h RPF) (Fig. 2f). This suggests that Dpp signaling is present in midguts prior to the onset of removal, which then decreases coincident with midgut degradation. Thus, it appears that Dpp signaling plays an important role in communication between the cells in the larval gut to prevent the premature degradation of this tissue.

Together, this suggests there is a complex role of Dpp signaling in the larval midgut that may function to coordinate midgut degradation. We propose that Dpp is expressed in the ISC/EB and the Dpp signal is received by the ECs to promote activation and nuclear localization of Mad. A similar role for Dpp has also been identified during spermatogenesis, where *dpp* expression in the somatic cells provides the Dpp ligand signal to the neighboring germ cells [27]. Activation of the Dpp pathway in ECs in the adult gut inhibits Dpp ligand expression [28], which is consistent with our findings of low levels of Dpp in larval ECs. Given the role of Dpp in communication between different cell/tissue types, it appears that Dpp signaling between the cells in the larval gut plays an important role in the correct timing of midgut removal.

### Dpp signaling prevents autophagy and larval midgut degradation

**Fig. 3:**
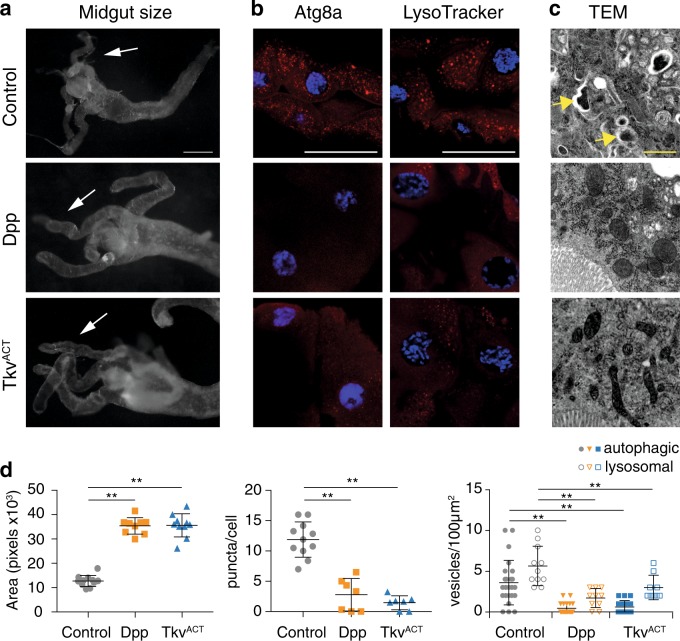
Dpp signaling blocks midgut removal and autophagy. **a** Morphology of control (*NP1-GAL4/+*), Dpp (*NP1-GAL4/+; UAS-dpp/+*), and Tkv^ACT^ (*NP1-GAL4/+; UAS-tkv*^*ACT*^*/+*) midguts from late third instar animals (−4 h RPF) shows enlarged midgut and gastric caeca (arrows). Scale bar represents 200 μm. **b** Autophagy induction detected by Atg8a puncta (red) and LysoTracker Red (red) in control midgut cells with low basal levels in Dpp and Tkv^ACT^ midguts at −4 h RPF. DNA is stained by Hoechst (blue). Scale bar represents 100 μm. **c** Representative TEM images from sections of midgut at −4 h PRF. Control cells possess autolysosomal structures (arrows), while Dpp and Tkv^ACT^ midgut cells lack structures. Scale bar represent 1 μm. **d** Quantitation of the gastric caeca size from (**a**) (average pixels ± SD) (^**^p *<* 0.0001, compared to control), autophagy puncta from (**b**) measured using ImageJ (average ± SD) (^**^p *<* 0.0001), and number of autophagic vesicles (solid shape indicates autophagosomes and autolysosomes) and lysosomes (outlined shape indicates lysosomes) from **c** (average ± SD) (^**^p *<* 0.0001)

Having uncovered a critical role of Dpp signaling for the survival of midgut cells we examined the effect of persistent Dpp signaling on the whole tissue. We found that Dpp signaling blocked larval midgut removal (Figs. 3a, d). Expression of Dpp or Tkv^ACT^ using the highly specific midgut driver, mex-GAL4, or an alternative midgut driver, NP1-GAL4, [29] resulted in larger midguts that failed to show gastric caeca contraction compared to the control that had begun to contract (Fig. 3a, d; Supplementary information, Fig. 2a). While the drivers are expressed during earlier gut development, the phenotype was not apparent until late third instar larval stage as there was no significant difference in the size of Dpp and Tkv^ACT^ midguts compared to controls from early third instar larvae (−16 to −8 h RPF) (Figure S2b). In addition, animals that expressed Dpp in the midgut did not undergo metamorphosis and died as late third instar larvae, while the *mex-tkv*^*ACT*^ animals died at a later stage as early pupae.

To further establish the critical period required for Dpp expression we used the temperature-sensitive Gal4 inhibitor, Gal80ts, to regulate Dpp expression from the NP1-GAL4 driver (referred to as NP1^ts^). At the permissive temperature (18 or 25 °C), Gal4 activity is blocked by Gal80ts, thus preventing Dpp or Tkv^ACT^ expression. Transfer of *NP1*^*ts*^ *>* *dpp* animals to the restrictive temperature (29 °C) inactivating Gal80ts following the third instar larval molt resulted in enlarged midguts and late larval or early pupal lethality (Supplementary information, Figure S2c). This indicates that the critical time for Dpp signaling in the midgut is just prior to the critical weight developmental checkpoint prior to nutrient independent growth. These findings support a new role for Dpp signaling in regulation of autophagy-dependent midgut degradation during metamorphosis.

The ecdysone-triggered midgut removal is dependent on autophagy induction [11, 30]. To determine if Dpp mediated blockade of midgut degradation was due to a loss of autophagy, we examined markers of autophagy. At the onset of midgut degradation the control midguts had induced autophagy with high levels of autophagy (Atg8a puncta) whereas the midguts-expressing Dpp and Tkv^ACT^ showed low basal levels of Atg8a puncta (Fig. 3b; Supplementary information, Figure S2d). LysoTracker staining has also been used in *Drosophila* to detect autophagy-associated lysosomal activity in the fat body and midgut [30, 31]. Consistent with low-Atg8a puncta, the Dpp and Tkv^ACT^ midgut cells also had reduced LysoTracker staining (Fig. 3b, d). Ultrastructural analysis showed that the Dpp and Tkv^ACT^ midguts lacked autolysosomal structures and contained more mitochondria (Fig. 3c, d), similar to the effect of blocking autophagy [32]. Together, these data indicate that autophagy induction is blocked by Dpp and Tkv^ACT^ expression in the midgut.

### Blocking Dpp signaling induces autophagy and midgut removal

**Fig. 4:**
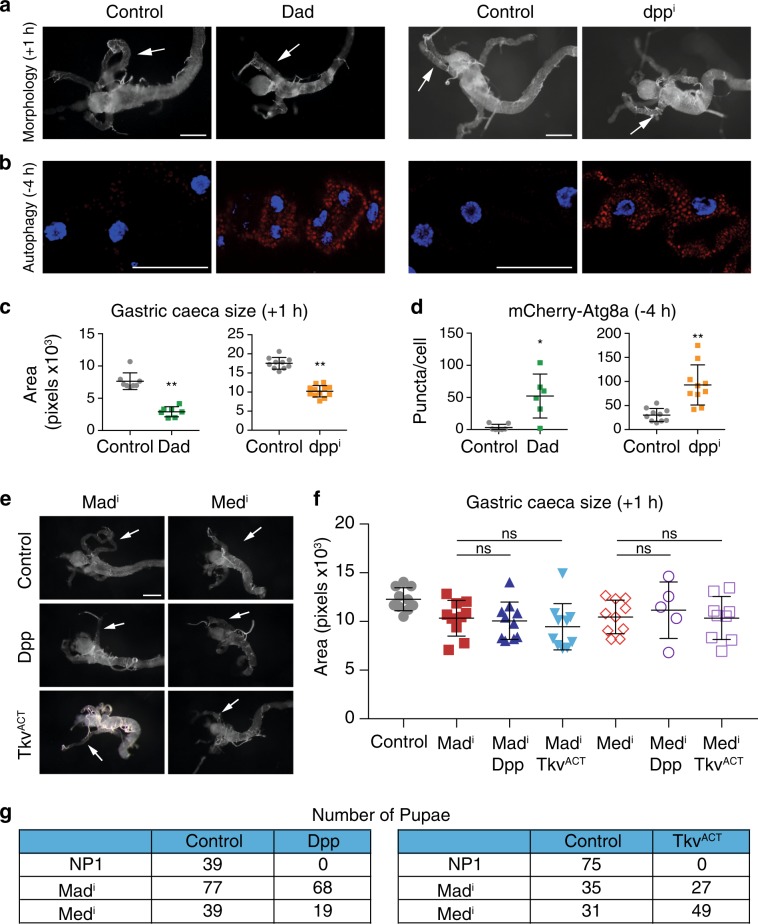
Blocking Dpp pathway induces premature autophagy and midgut removal. **a** Morphology of control (*NP1-GAL4/+; pmCherry-Atg8a/+*), Dad-expressing (*NP1-GAL4/UAS-Dad; pmCherry-Atg8a/+*) and *dpp*^*i*^ (*NP1-GAL4/UAS-dpp*^*RNAi*^*; pmCherry-Atg8a/+*) midguts from +1 h shows smaller midgut and more contracted gastric caeca (arrows). Scale bar represents 200 μm. **b** Autophagy monitored by mCherry-Atg8a puncta (red) shows that expression of Dad and knockdown of *dpp* promotes premature induction of autophagy in the larval midguts at −4 h RPF (arrows). Scale bar represents 50 μm. **c** Quantitation of gastric caeca size at +1 h RPF from **a** (average pixels ± SD) (^**^p < 0.0001). **d** Quantitation of puncta at −4 h RPF from **b** (average ± SD) (^*^p *=* 0.003, ^**^p *=* 0.0003). **e** Morphology at +1 h RPF shows contracted gastric caeca (arrows) from knockdown of *Mad*^*i*^ (*NP1* *>* *Mad*^*RNAi*^) and *Med*^*i*^ (*NP1* *>* *Med*^*RNAi*^) with the co-expression of Dpp or Tkv^ACT^. Scale bar represents 200 μm. **f** Quantitation of gastric caeca size from (E) (average pixels ± SD). (ns, not significant). **g** Number of pupae from combined *Mad* or *Med* knockdown with either expression of Dpp or Tkv^ACT^

To examine the effect of blocking Dpp signaling we expressed the inhibitory Smad, Dad, in the midgut. This resulted in both premature midgut degradation, as evidenced by shorter gastric caeca (+1 h RPF) and premature induction of autophagy (−4 h RPF) (Fig. 4a–d). Similarly, the knockdown of *dpp* in the midgut resulted in premature gastric caeca contraction and early autophagy induction (Fig. 4a–d). Similar results were seen using another independent *dpp* knockdown line. The phenotype observed by knockdown of *dpp* was less pronounced compared to the phenotype observed with the expression of Dad, and this may be due to the incomplete knockdown of *dpp* (Supplementary information, Figure S3a).

To determine if the role of Dpp signaling on autophagy-dependent midgut removal was due to downstream Dpp signaling components, we examined the effects of combining the knockdown of Smads, *Mad* and *Med*, (Supplementary information, Figure S3b) with the expression of Dpp or Tkv^ACT^. The knockdown of *Mad* and *Med* combined with Dpp or Tkv^ACT^ showed strong suppression following induction of midgut PCD (+1 h RPF), while the Dpp or Tkv^ACT^ animals developmentally arrest prior to this stage (Fig. 4e, f). This rescue was not observed by knockdown of independent pathway genes (e.g., Figs 7c–e). Thus, *Mad* and *Med* knockdown was sufficient to rescue both midgut morphology and the larval lethality caused by *NP1* *>* *dpp* or *NP1* *>* *tkv*^*ACT*^ (Fig. 4g). These results are consistent with Dpp signaling through the canonical signaling pathway blocking midgut degradation.

### Dpp modulates the expression of genes involved in autophagy and ecdysone response

**Fig. 5:**
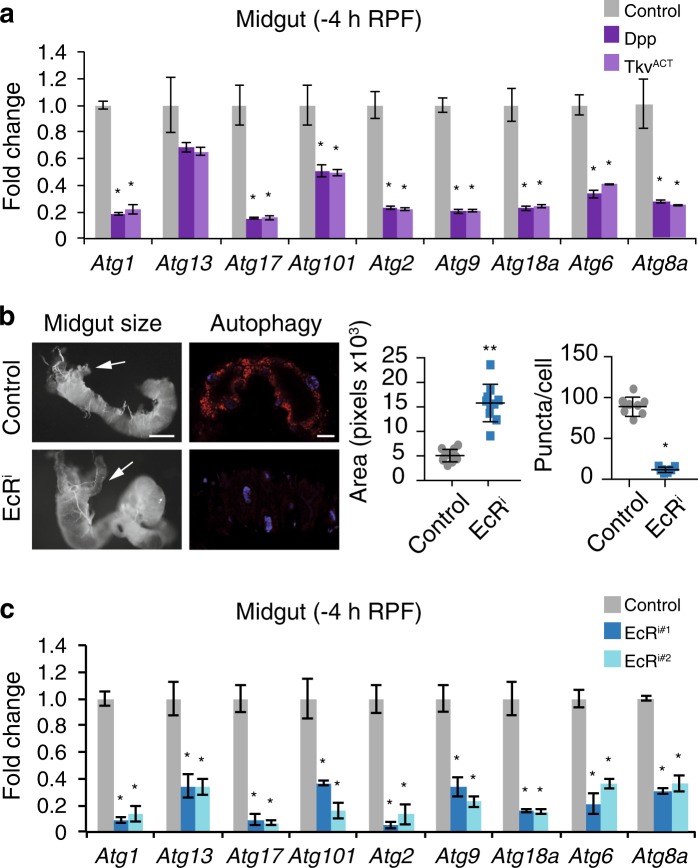
Dpp signaling impairs ecdysone dependent signaling. **a** *Atg* gene expression is reduced in larval midguts-expressing Dpp or Tkv^ACT^. Transcript levels were measured by qRT-PCR in control, Dpp and Tkv^ACT^ larval midguts at −4 h RPF. Data are from 3 experiments, with 20 midguts per sample (average ± SEM) (^*^p < 0.05). **b** EcR knockdown blocks autophagy and midgut histolysis. Morphology of control (*NP1-GAL4/+*) and *EcR*^*i*^ (*NP1-GAL4/+; UAS-EcR*^*RNAi*^*/+*) midguts at +4 h RPF show enlarged midgut and gastric caeca (arrows). Scale bar represents 200 μm. Quantitation of the gastric caeca size (average pixels ± SD) (^**^p *<* 0.0001). Autophagy monitored by mCherry-Atg8a puncta (red) from control and *EcR*^*i*^ midguts from +2 h RPF. DNA is stained by Hoechst (blue). Scale bar represents 50 μm. Quantitation of puncta at +2 h RPF (average ± SD) (^*^p *=* 0.003). **c** *Atg* gene expression is reduced in larval midguts in two independent *EcR* RNA*i* lines. Transcript levels were measured by qRT-PCR in contro1 (NP1/+), *NP1* *>* *EcR*^*i*^ #1 and *NP1* *>* *EcR*^*i*^ #2 larval midguts at −4 h RPF (average ± SEM) (^*^p < 0.05)

The multistep autophagy pathway is regulated by a number of distinct *Atg* genes that are upregulated during autophagy-dependent midgut removal [11, 30]. To determine if the reduced autophagy and block in midgut removal by Dpp signaling was due to lower expression of genes essential for autophagy we examined the expression of several *Atg* genes in both Dpp or Tkv^ACT^ midguts (Fig. 5a). The expression of several key *Atg* genes required for midgut degradation was significantly reduced in both Dpp and Tkv^ACT^ expressing midguts (Fig. 5a). These observations further support the role of Dpp in preventing autophagy and *Atg* gene expression.

The requirement for ecdysone in the removal of the obsolete larval tissue during metamorphosis is well established [33, 34]. Ecdysone signaling, mediated by the heterodimeric receptor EcR/Usp, regulates the transcription of target genes directly or through early (or primary) regulatory genes encoding transcription factors [7, 9, 10]. During salivary gland histolysis, the expression of several autophagy and apoptosis genes increases in response to ecdysone, in part due to the direct binding of EcR/Usp to the promoters [35, 36]. To establish the requirement of ecdysone signaling in regulation of autophagy during midgut degradation, the effect of *EcR* knockdown in the midgut was examined. The knockdown of *EcR* in the midgut blocked autophagy, delayed midgut removal (Fig. 5b; Supplementary information, Figure S4a), and resulted in decreased expression of all *Atg* genes examined (Fig. 5c).

### Dpp signaling in the midgut blocks the developmental ecdysone response

**Fig. 6:**
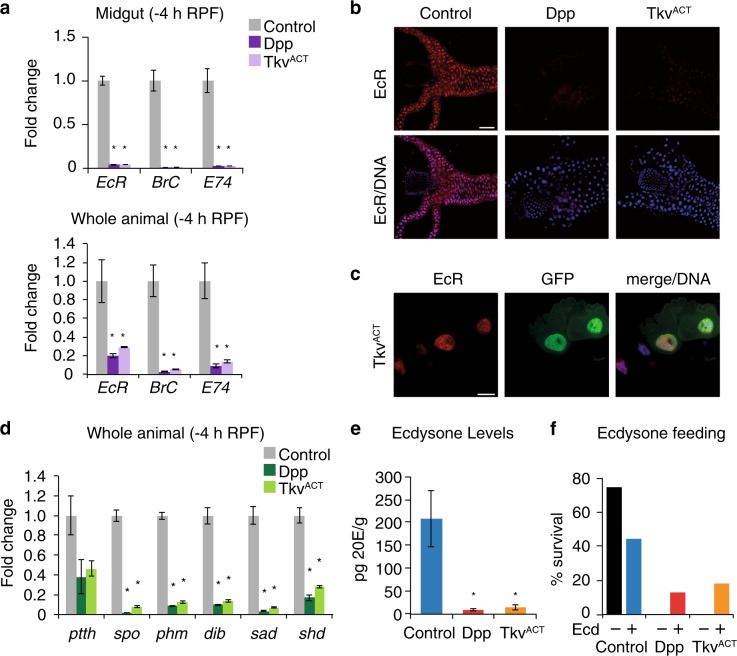
Dpp signaling blocks ecdysone production. **a** The levels of *EcR, Br-C*, and *E74* are reduced in larval midgut and in whole larvae expressing Dpp or Tkv^ACT^ in the midgut. Transcript levels were measured by qRT-PCR from control, *NP1* *>* *dpp* and *NP1* *>* *tkv*^*ACT*^ larval midguts at −4 h RPF. Data are from 3 experiments, with 20 midguts per sample (average ± SEM) (^*^p < 0.01). **b** Control, *NP1* *>* *dpp* and *NP1* *>* *tkv*^*ACT*^ midguts from larvae at −4 h RPF stained with EcR antibody (red) show dramatically reduced EcR. DNA is stained by Hoechst (blue). Scale bar represents 100 μm. **c** Tkv^ACT^-expressing cell (*hsFLP; pmCherry-Atg8a/+; Act* *>* *CD2* *>* *GAL4, UAS-nlsGFP/UAS-tkv*^*ACT*^) marked by GFP (green) has similar levels of EcR (red) compared to the neighboring control cells from −4 h RPF gastric caeca. DNA is stained by Hoechst (blue). Scale bar represents 25 μm. **d** Transcript levels of *ptth*, *spo, phm, dib, sad, shd*, and *ecd* were measured by qRT-PCR from control (*NP1-GAL4/+*), *NP1* *>* *dpp* and *NP1* *>* *tkv*^*ACT*^ late third instar larvae (−4 h RPF). Data are from three experiments, with three larvae per sample (average ± SEM) (^*^p < 0.001). **e** The ecdysone titer from whole late third instar larvae (−4 h RPF) expressing Dpp *(NP1* *>* *dpp)* or Tkv^ACT^ *(NP1* *>* *tkv*^*ACT*^*)* in the midgut. Data are from 3 experiments, with a minimum of 15 larvae per sample (average ± SD) (^*^p < 0.05, compared to control). **f** Survival of *NP1* *>* *dpp* or *NP1* *>* *tkv*^*ACT*^ to pupal stage following feeding of food supplemented with 20E or vehicle (ethanol)

With the essential role of ecdysone in midgut removal and the requirement of *EcR* for the expression of *Atg* genes, it is possible that Dpp signaling represses *EcR* and, thus its downstream primary response genes to alter *Atg* gene expression during midgut removal. Examination of the expression of *EcR* and primary response genes, *BrC* and *E74* by qRT-PCR revealed a dramatic decrease in the transcript levels of these genes in Dpp and Tkv^ACT^-expressing midguts (Fig. 6a). The levels of *EcR* and primary response genes were also decreased in whole animals expressing Dpp and Tkv^ACT^ in the midgut (Fig. 6a). Furthermore, the levels of EcR in the nucleus of midgut cells was dramatically reduced in response to Dpp or Tkv^ACT^ expression in the midgut (Fig. 6b; Supplementary information, Figure S4b). Interestingly, the transcript levels of *EcR* in *mex* *>* *tvk*^*ACT*^ were similar to the control (Supplementary information, Figure S4c), yet the expression of EcR target genes was reduced, consistent with the weaker phenotype of this line. However, in clones of cells expressing Tkv^ACT^ in the midgut there was no decrease in EcR staining (Fig. 6c). This suggests that the reduced EcR in the midguts is not simply due to Dpp expression in this tissue, but that a signal is produced from the midguts with maintained Dpp signaling that blocks the EcR response. It also implies that the developmental arrest due to Dpp expression in the midgut is not a consequence of the enlarged tissue. This is in agreement with the knockdown of other genes (e.g., *Atg1* [11] or *EcR* that block autophagy) that result in enlarged midguts due to delayed degradation yet these animals undergo metamorphosis with no developmental block observed.

The transcriptional activity of EcR/Usp requires ecdysone binding and *EcR* expression is directly induced by ecdysone [37]. Given the reduced EcR signaling in animals expressing Dpp and Tkv^ACT^ in the midgut it was possible that this was due to a reduction in ecdysone levels. The production of ecdysone occurs in the prothoracic glands (PG), and requires a number of biosynthetic enzymes. Following its release from the PG ecdysone is taken up by target tissues where it is converted into the active form 20E [38]. Prothoracicotropic hormone stimulates ecdysone production in the PG and requires the function of several biosynthesis genes including *spook* (*spo*), *phantom* (*phm*), *disembodied* (*dib*), *shadow* (*sad*), and *shade* (*shd*). To determine if ecdysone production was disrupted we examined the levels of ecdysone biosynthesis genes in whole larvae that expressed Dpp and Tkv^ACT^ in the midgut. This showed significantly reduced expression of several ecdysone biosynthesis genes (Fig. 6d, Supplementary information Figure S4d). Consistent with this, late third instar larvae expressing Dpp and Tkv^ACT^ in the midgut had significantly reduced ecdysone titer (Fig. 6e). These results indicate that maintaining Dpp signaling in the midgut blocks the developmental ecdysone response. Thus, the transcriptional activation of genes required for ecdysone production is blocked in response to activation of the Dpp pathway in the midgut impeding ecdysone production. In support of this we found the block in metamorphosis in *NP1* *>* *dpp* and *NP1* > *tkv*^*ACT*^ animals could be partially rescued by feeding larvae with 20E (Fig. 6f). Thus, reduced ecdysone production contributes to the developmental arrest, and Dpp produced by the midgut plays a key role in regulating the correct timing of ecdysone biosynthesis. This is consistent with the down-regulation of Dpp activity indicated by the decrease in nuclear pSmad in the midgut prior to metamorphosis (Fig. 2f).

### Dpp blocks the production of ecdysone

**Fig. 7:**
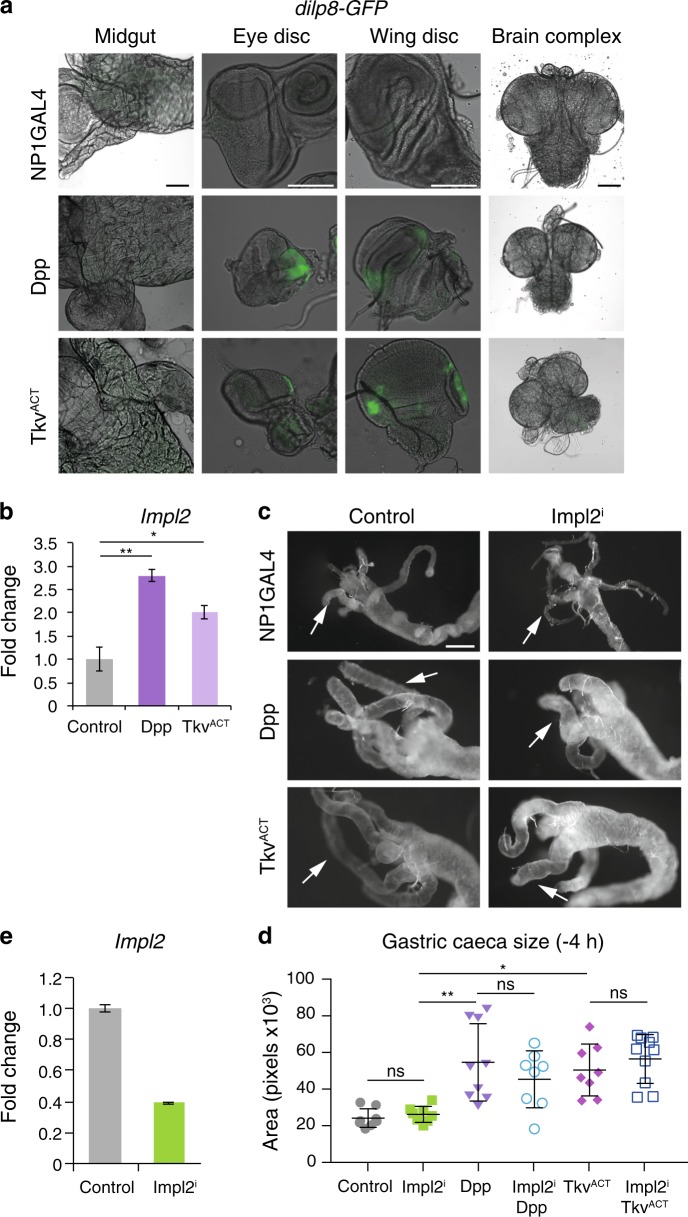
Dpp dependent block in ecdysone is not mediated by Dilp8 or Impl2. **a** Dilp8-GFP expression is undetectable in the midgut and brain complexes with modest GFP detected in eye and wing disc from *NP1* *>* *dpp*, *NP1* *>* *tkv*^*ACT*^. Scale bars, 100 µm. **b** The level of *Impl2* transcripts measured by qRT-PCR from *NP1* *>* *dpp* and *NP1* *>* *tkv*^*ACT*^ larvae was increased compared to the control at −4 h RPF. Data are from three experiments, with three larvae per sample (average ± SEM) (^**^p *<* 0.01, ^*^p < 0.05 compared to control). **c** Knockdown of *Impl2*^*i*^ (*NP1-GAL4/UAS-Impl2*^*RNAi*^), did not suppress the phenotype of *NP1* *>* *dpp* and *NP1* *>* *tkv*^*ACT*^ midguts at −4 h RPF by comparing contraction of the gastric caeca (arrows). Scale bar represents 200 μm. **d** Quantitation of gastric caeca size in (B) (average pixels ± SD). (^**^p *<* 0.001, ^*^p < 0.01 and ns, not significant). **e** Quantitation by qRT-PCR of the level of RNA*i* mediated knockdown of *Impl2* (average ± SEM)

As the animals with maintained Dpp signaling in the midgut resulted in a block in ecdysone production we investigated the potential signal from the Dpp-expressing midgut that prevents ecdysone production in the PG. The insulin/relaxin-like peptide Dilp8 acts to ensure the coordination of organ growth with animal maturation [39, 40]. During larval development abnormally growing imaginal discs secrete Dilp8 to delay metamorphosis by inhibiting ecdysone biosynthesis. To examine if upregulation of Dilp8 is contributing to the block in ecdysone production from *NP1* > *dpp* or *NP1* > *tkv*^*ACT*^, we examined a GFP reporter of Dilp8. Expression of GFP could be detected in the wing and eye imaginal disc, but could not be detected in the midgut or the brain and ring gland complex (Fig. 7a). The low levels of Dilp8 detected in the *NP1* > *dpp* and *NP1* > *tkv*^*ACT*^ animals that fail to pupate are not consistent with the ectopic expression of Dilp8 in imaginal discs that has been shown to delay pupariation by 2–3 days [39, 40], suggesting that Dilp8 alone is unlikely to be responsible for blocking ecdysone production preventing metamorphosis.

The insulin/IGF antagonist, Imaginal morphogenesis protein-Late 2 (ImpL2) acts as a sensor of the nutritional state of larvae and coordinates dietary information and ecdysone production to modulate developmental transitions [41]. In the adult, ImpL2 is secreted from intestinal tumors that mediates organ wasting [42, 43]. We detected increased levels of *Impl2* in animals expressing Dpp and Tkv^ACT^ in the midgut (Fig. 7b). To investigate if the block in ecdysone production in the PG was due to the expression and secretion of ImpL2 from the midgut we ablated *Impl2* in the Dpp or Tkv^ACT^ larval midgut (Fig. 7c–e). However, this did not rescue the midgut morphology or the developmental arrest, suggesting that Impl2 is not secreted from the midgut (Fig. 7c, d).

**Fig. 8:**
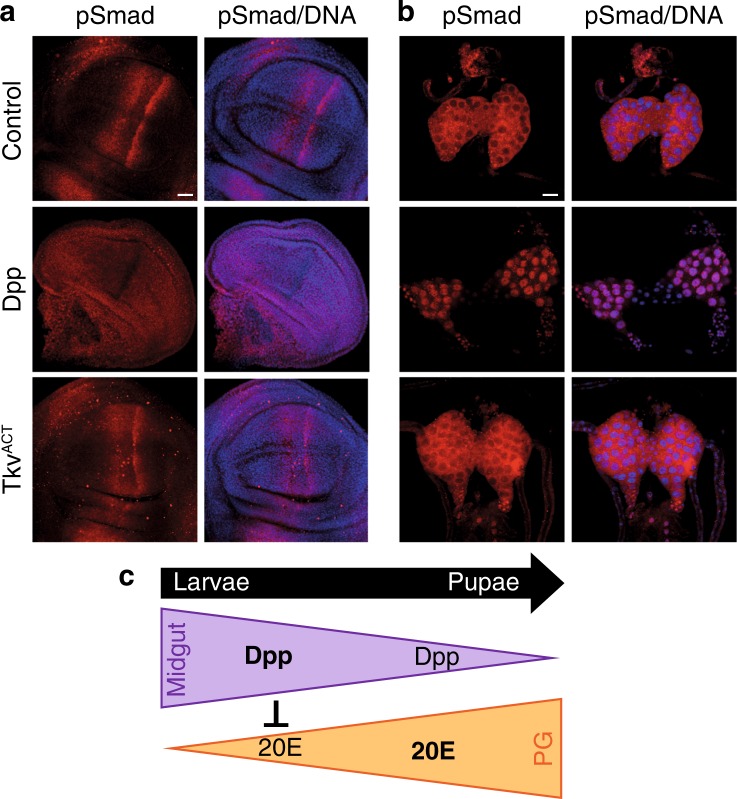
Block in ecdysone production due to Dpp expressed in the midgut. **a** Spatial localization of phospho-Smad (red) and DNA (blue) in wing discs from control*, mex* *>* *dpp* and *mex* *>* *tkv*^*ACT*^ larvae at −4 h RPF showing loss of the distinct pattern of pMad in *mex* *>* *dpp*. Scale bar represents 50 μm. **b** Nuclear localization of phospho-Smad (red) and DNA (blue) in ring glands from control*, mex* *>* *dpp* and *mex* *>* *tkv*^*ACT*^ larvae at −4 h RPF. Scale bar represents 50 μm. **c** Model of the proposed crosstalk between Dpp and ecdysone signaling during metamorphosis

An alternative possibility was that Dpp from midgut directly contributed to block ecdysone production by activating Dpp pathway in the PG. A recent study has shown that Dpp expressed in the fat body is secreted into the hemolymph resulting in perturbed Dpp signaling in the wing disc [44]. We investigated the potential for Dpp expressed in the midgut to promote Dpp signaling in peripheral tissue. Compared to the distinct pattern of activated Mad localization in the control wing disc, in animals expressing Dpp in the midgut homogeneous Dpp signaling in the wing disc was detected by the altered pattern of activated Mad staining (pSmad, Fig. 8a). This was in contrast to animals expressing Tkv^ACT^ in the midgut, where the pattern of Mad localization was similar to control discs (Fig. 8a). More importantly, strong nuclear Mad localization was detected in the prothoracic gland of animals expressing Dpp in the midgut but the localization remained cytoplasmic in response to Tkv^ACT^ expression in the midgut (Fig. 8b). This suggests that there is a signal produced from midguts that express Dpp that can signal to other peripheral tissue including the PG.

## Discussion

Our data identifies a previously unreported role for Dpp signaling as a critical regulator of autophagy-dependent midgut degradation. We show that canonical Dpp signaling prevents midgut degradation, while blocking Dpp signaling prematurely induces autophagy and midgut removal. In mosaic clones, cells expressing Tkv^ACT^ have delayed cell size contraction and reduced autophagy during midgut PCD, whereas cells expressing Dad show premature cell size reduction and increased autophagy. Importantly, *Mad*^*8–2*^ and *tkv*^*5*^ mutant cells undergo rapid removal from the larval midgut. The unique morphology of dying *Mad*^*8–2*^ and *tkv*^*5*^ mutant cells also provides the first visual glimpses into the mechanism of autophagy-mediated cell death, as the cell contents appear to be rapidly degraded by lysosomal enzymes leaving behind cell corpses which appear as holes in the midgut tissue.

The spatial and temporal control of midgut growth and PCD involves the integration of several major inputs, including growth and hormonal signals. Down-regulation of growth signaling is a prerequisite for autophagy-dependent midgut PCD as is the input from hormonal cues. Interestingly, in the PG signaling pathways InR and Torso, acting via PI3K and Ras, respectively, are important for the upregulation of ecdysone biosynthesis genes and require BMP/activin signaling to ensure their expression [45]. Our findings now reveal that Dpp signaling prevents autophagy and ecdysone production, and it will be important to examine the coordination between Dpp, InR, and Ras signaling in the midgut.

The removal of the larval midgut occurs when the larvae stop feeding, and our data suggest that the midgut plays an important role in the coordination of growth and hormonal cues to establish a developmental checkpoint. Our studies also indicate that Dpp has dual roles, locally in the midgut during ecdysone-mediated PCD at the onset of metamorphosis and upstream as a regulator of ecdysone production from PG (Fig. 8c). Our data suggest that Dpp expressed in the midgut produces a signal responsible for the developmental arrest and block in ecdysone production. One possible model is that Dpp acts a sensor; if developmental cues are compromised Dpp activity results in a feedback to block ecdysone production, preventing midgut histolysis and metamorphosis thereby establishing a developmental transition checkpoint. Future studies to dissect out this important function of Dpp activity in the midgut will need to be undertaken to establish how the long-range signal is established and how this leads to the developmental checkpoint.

It is interesting that Dpp has distinct roles in the gut at various developmental stages. During embryonic gut formation Dpp is required in a complex feedback, both within and between tissue layers, to maintain the correct patterning of homeotic gene expression during midgut morphogenesis [46, 47]. In the *Drosophila* adult gut, Dpp has multiple complex roles in the regulation of ISCs in both an autocrine and paracrine mode [48–53]. Additionally, the TGF-β/activin pathway acts as a carbohydrate-sensing mechanism in the adult midgut to regulate digestive enzyme expression [54]. After more than 20 years exploring the role of Dpp as a tissue organizer, our studies have now uncovered another key role for Dpp, required for hormone-mediated autophagy-dependent midgut removal. The involvement of TGF-β signaling as a regulator of autophagy has also been reported in *Caenorhabditis elegans* [55, 56], thus suggesting that the function of Dpp may be conserved.

## Materials and methods

### Fly stocks

The midgut driver *P{GawB}Myo31DF*^*NP0001*^ referred to as *NP1-GAL4*, was obtained from the *Drosophila* Genetic Resource Center (Kyoto, Japan), and *mex-GAL4* from R. Burke (Monash University, Vic., Australia). The following stocks were from the Bloomington *Drosophila* Stock Center (Bloomington, IN, USA) including RNAi lines from the Transgenic RNAi Project (http://www.flyrnai.org): *w*^*1118*^, *UAS-dpp* (w*; UAS-dpp.S 42B), *UAS-tkv*^*ACT*^ (w*; UAS-tkv.Q253D/TM3, Sb^*1*^ *Ser*^*1*^), *EcR*^*i*^ *#2* (*w*^*1118*^*; UAS-EcR-RNAi s104*), *Mad*^*8-2*^ (w*; Mad^*8-2*^ *FRT-40A/CyO*) *dpp-GAL4* (w*; wg^*Sp-1*^*/CyO; P{GAL4-dpp.blk1}40C.6/TM6B, Tb*^*1*^), *omb-lacZ, dpp-lacZ* (*cn*^*1*^*; P{dppshv-lacZ.RD2}RD2, ry*^*506*^), *dpp*^*i*^ (*y*^*1*^ *v*^*1*^*; P{TRiP.JF01371}attP2*), *Mad*^*i*^ (*y*^*1*^ *v*^*1*^*; P{TRiP.JF01263}attP2*), *Med*^*i*^ (*y*^*1*^ *v*^*1*^*; P{TRiP.JF02218}attP2*), *impl2i y*^*1*^ w*; {MiMIC}Ilp8^*MI00727*^ and *UAS-GFP* (*w*^*1118*^*; UAS-EGFP 5a.2*). The *ImpL2*^*i*^ (*P{KK112218}VIE-260B*) line was from Vienna *Drosophila* RNAi Center, *EcR*^*i*^ *#1* (*UAS-EcR-RNAi*) line was from NIG-Fly*, ywhsFlp; pmCherry-Atg8a; Act* > *CD2* > *GAL4, UAS-nlsGFP/TM6B* from E. Baehrecke*, hsFLP; Ubi-GFP FRT-40A*, *tkv*^*5*^ *FRT-40A /CyO*, and *UAS-Dad* from H. Richardson (La Trobe University, Vic., Australia), and *tub-Gal80ts* from G. Hime (Melbourne University, Vic., Australia. The control was *w*^*1118*^ crossed to the driver line. Verification and quantitation of the RNA*i* lines was determined by qRT-PCR from late third instar larvae (−4 h RFP) from 20 midguts/sample in triplicate. All flies were maintained and crossed performed at 25 °C on cornmeal, molasses, and yeast medium.

### Larval staging, midgut morphology analysis, and 20E feeding

Larvae were staged by feeding on food containing 0.05% bromophenol blue and transferred as wandering third instar larvae into a petri dish lined with moist Whatmann paper to monitor for gut clearance as visualized by loss of blue in the gut [57]. For temperature shift experiments, crosses were allowed to lay eggs in 4 h batches and aged at 18 °C prior to shifting to 29 °C. For morphological analysis, a minimum of 10 midguts from appropriately staged animals were dissected in PBS, fixed in 4% formaldehyde/PBS and imaged using a stereozoom microscope (Olympus, Tokyo, Japan). Measurements of the gastric caeca size from images was done using Photoshop (Adobe, San Jose, CA, USA) magnetic lasso tool and the histogram function used to determine pixels included in the area as previously described [12]. For ecdysone feeding larvae were collected as first instar and placed in groups of 10 in 1.5 ml tubes containing 200 μl standard food supplemented with 20E (Sigma-Aldrich, St. Louis, MO, USA) dissolved in ethanol (0.5 mg/ml), capped with cotton wool. Control larvae were fed with standard food mixed with the same volume of ethanol. A total of 300 larvae were collected from each genotype.

### Live mCherry and LysoTracker imaging

To assay for mCherry-Atg8a, a minimum of 10 midguts were dissected from appropriately staged animals in PBS with Hoechst 33342 (Sigma-Aldrich) to stain DNA and imaged immediately without fixation using a Zeiss LSM 700 confocal (Detmold Imaging Core Facility, SA Pathology, Adelaide, SA, Australia). The images were quantitated using ImageJ to count puncta with a size larger than two pixels and represented as the average puncta per cell. To assay for LysoTracker, a minimum of 10 midguts were dissected in PBS with 1 µM LysoTracker Red and Hoechst 33342. The images were quantitated using ImageJ to count puncta with a size larger than 30 pixels, and represented as the average puncta per cell.

### Induction of cell clones and chloroquine feeding

To induce Tkv^ACT^ or Dad clones, *ywhsFlp; pmCherry-Atg8a; Act* > *CD2* > *GAL4, UAS-nlsGFP/TM6B* was crossed to *UAS-tkv*^*ACT*^/*TM6B* or *UAS-Dad* and 1-day-old embryos were heat shocked at 37 °C for 5 min. Cell size was quantitated using ImageJ [58]. To generate mutant clones *hsFlp; ubi-GFP FRT-40A* was crossed to *Mad*^*8–2*^ *FRT-40A/CyO actGFP* and *tkv*^*5*^ *FRT-40A/CyO actGFP* mutant allele lines on the same chromosome as the FRT. The cross was allowed to lay for 8 h then embryos were heat shock at 37 °C for 60 min. For chloroquine feeding of larvae, chloroquine diphosphate salt (CQ, Sigma) was dissolved in water (25 mg/ml), and added to standard food to a final concentration of 2.5 mg/ml. Control larvae were fed with standard food mixed with the same volume of PBS.

### Immunohistochemistry

Midguts of the required genotype were dissected from appropriately staged animals in PBS and fixed in 4% paraformaldehyde in PBS with phosphatase inhibitors (1:100) (Sigma, P5726) for 20 min at room temperature as described [31]. Primary antibodies used were rabbit anti-Phospho-Smad 1/5 (Ser463/465) (1:500) (Cell Signaling, 9516, Danvers, MA, USA), rabbit anti-GABARAP1, referred to as ATG8a, (1:200) (Abcam, Cambridge, MA, USA), mouse anti-EcR B1(1:400) (Development Studies Hybridoma Bank, DSHB), anti-prospero (1:10) (DSHB), rabbit anti-β-galactosidase (1:2000) (MP Biomedicals, Solon, OH, USA), and rabbit anti-GFP (1:500) (Abcam). Secondary antibodies were anti-rabbit Alexa-FLUOR 568 and anti-mouse Alexa-FLUOR 488 (Molecular Probes, Eugene, CA, USA), and Hoechst 33342 (Sigma-Aldrich) was used to detect DNA. The samples were imaged using a Zeiss LSM 700 confocal microscope.

### X-gal staining

The *lacZ* activity was detected using standard X-Gal (5-bromo-4-chloro-3-indolyl-β-d-galactopyranoside) staining procedure. Briefly, midguts from larvae were dissected in PBS, fixed in 0.8% glutaraldehyde in PBS for 15 min, washed in PBS and stained in 0.2% X-gal in staining buffer (10 mM NaH_2_PO_4_/Na_2_HPO_4_ pH7.2, 1 mM MgCl_2_, 5 mM K_3_Fe(CN)6, 5 mM K_4_Fe(CN)6, 0.4% SDS) at room temperature.

### Transmission electron microscopy

Midguts from appropriately staged larvae were dissected in 1×PBS then fixed in 1.25% glutaraldehyde, 4% sucrose, 4% paraformaldehyde in PBS for 30 min at room temperature. Samples were then washed with 4% sucrose in PBS, postfixed in 1% osmium tetroxide for 1 h, dehydrated, treated with propylene oxide, and infiltrated for embedding in resin as described [31]. Ultrathin sections were cut on grids, stained for 2 min with 4% uranyl acetate in 25% ethanol and 2 min in Reynold’s lead citrate before examination using Tecnai G2 Spirit TEM (Adelaide Microscopy).

### Confocal imaging

Confocal images were obtained using Zeiss LSM 700 or Zeiss LSM 800 inverted confocal microscope with Argon ion 488 nm (14 mw) and Green HeNe 543 nm (1.5 mw) lasers and a 40 × UPLAPO (NA = 1.2 water) objective. The dual labeled samples were imaged with two separate channels (PMT tubes) in a sequential setting. Green fluorescence was excited with an Ar 488 nm laser line and the emission viewed through a HQ515/30 nm narrow band barrier filter in PMT1. Red fluorescence was excited with a HeNe 543 nm laser line and the emission viewed through a long pass barrier filter (E570LP) in PMT2 (Detmold Facility). On LSM 700, Zen grey was used to capture the images. On LSM 800, Airyscan detector was used, pictures were captured and Airyscan processed using Zen blue. Images were then processed using Photoshop (Adobe).

### Quantitative real-time PCR (qRT-PCR)

Total RNA was isolated from 20 midguts/sample or 3 whole larvae/sample in triplicate for each sample using TRIzol reagent (Invitrogen). cDNA was synthesized using High Capacity cDNA Reverse Transcription Kit (Applied Biosciences, Life Technologies, Carlsbad, CA, USA) and random or oligo dT primers (Geneworks, Thebarton, SA, Australia), with 1 μg of total RNA. qRT-PCR was performed on a Qiagen Rotor-Gene Q using RT [2] SYBR Green qPCR MasterMix (Qiagen, Valencia, CA, USA) as per the manufacturer’s instructions. Reactions were performed in triplicate using three independent biological samples and the transcript levels were normalized using *rp49* as the reference gene. Data were analyzed using either the comparative CT method with optimized efficiencies for Target and Reference genes or using the Q-Gene software with standard curves [59], and samples on the same graph were run simultaneously. Primers for *Atg* genes are described [11, 30], and primers used are as follows:

*dpp* F 5′-gccaacacagtgcgaagtt; R 5′-cgcgtgatgtcgtagacaag *Mad* F 5′-ctgagcaacgtgaacaggaa; R 5′-gatggaatccgtggtggtag *Med* F 5′-accctcacctacacgcagtc; R 5′-tatgcgatggagcaccagta*Br-C* F 5′-acaacaacagccccgactt; R 5′-gcttgtcgctgatggagatt *E74* F 5′-ccacaatctgcttagcggc; R 5′-ctgggcggaaatgaacctgt *EcR* F 5′-ctcagctgcaaggtcaactg; R 5′-ccatgtattcgctgctcgta *ptth* F 5′-ggctgcgactgcaaagttac; R 5′-ccacgaataggggtgatcgg *spo* F 5′-tatctcttgggcacactcgctg; R 5′-gccgagctaaatttctccgctt *phm* F 5′-ggatttctttcggcgcgatgtg; R 5′-tgcctcagtatcgaaaagccgt *dib* F 5′-tgccctcaatccctatctggtc; R 5′-acagggtcttcacacccatctc *sad* F 5′-ccgcattcagcagtcagtgg; R 5′-acctgccgtgtacaaggagag *shd* F 5′-cgggctactcgcttaatgcag; R 5′-agcagcaccacctccatttc *Impl2* F 5′-tacaagtgcatagcccgcaa; R 5′-ttcacgcatcttcgaaggca

### Measurement of ecdysone titer

Late third instar larvae were collected from control, Dpp and Tkv^ACT^ (minimum of 15 larvae/sample in triplicate) snap frozen in liquid nitrogen and stored at −80 °C. The weighed samples were homogenized in 300 µl of methanol, centrifuged at 16,000×*g* for 20 min twice with the combined methanol supernatants dried prior to resuspending in 50 µl enzyme immunoassay buffer (Cayman Chemicals, Inc., USA). The ecdysteroid levels were measured as 20E equivalents and the assay was performed using 20E antiserum, 20E-acetylcholinesterase Tracer and Ellman reagent (Cayman Chemicals) according to the manufacture’s protocol. Briefly, 50 µl of the standards and diluted samples were incubated with 50 µl of tracer and antiserum in antirabbit IgG coated ELISA plates overnight, washed five times and developed by adding 200 µl of Ellman’s reagent. The absorbance was read at 405 nm on Spectrophotometer (Fluostar) and all assays were performed in triplicate.

### Statistical analysis of data

Student’s *t* test, two-tailed equal variance was used for statistical analysis to compare two groups and data are expressed as mean ± standard deviation (SD) or mean ± standard error of the mean (SEM), as appropriate. *P* < 0.05 was considered significant. For multiple analyses an ordinary one-way analysis of variance (ANOVA) with Tukey’s multiple comparisons test was performed using Prism (GraphPad Software) and data are expressed as mean ± SD. There was no statistical method used to predetermine sample size. The experiments were not randomized and no samples were excluded. There was no blind allocations of the experiments or interpretation of results.

## Electronic supplementary material

Supplementary Data
