# Supplementary material for: Dpp regulates autophagy-dependent midgut removal and signals to block ecdysone production
Source: Cell Death Differ. 2018 Jun 29;26(4):763–78. doi: 10.1038/s41418-018-0154-z (PMC6460390; doi:10.1038/s41418-018-0154-z)
Supplement: Supplementary file 1 — Supplementary Data [file 41418_2018_154_MOESM1_ESM.pdf]

Supplementary Information:

Dpp regulates autophagy-dependent midgut removal and signals to block ecdysone production

Donna Denton, Tianqi Xu, Sonia Dayan, Shannon Nicolson and Sharad Kumar

Supplementary Figures S1-S4.

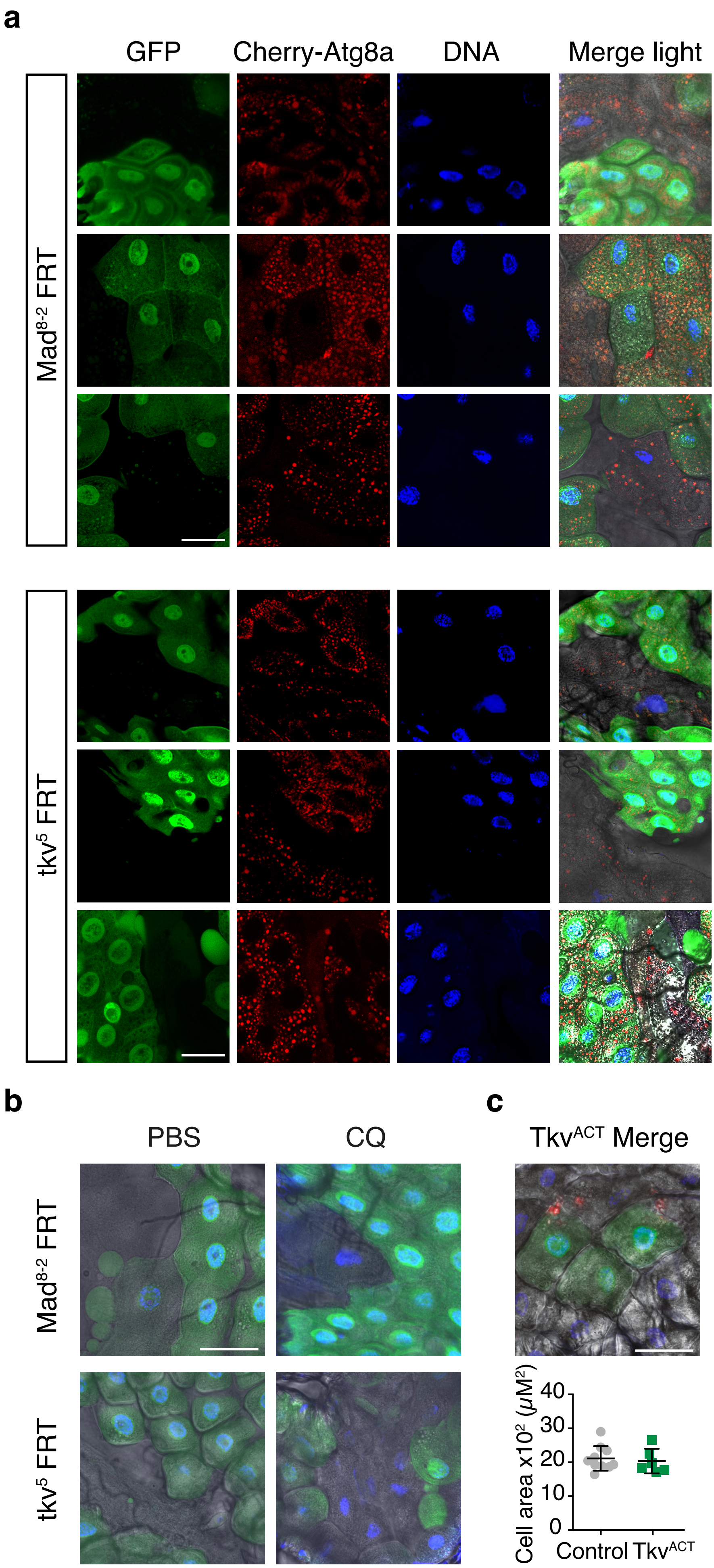
a
GFP
Cherry-Atg8a
DNA
Merge light
Mad8-2 FRT
tkv5 FRT
b
PBS
CQ
Mad8-2 FRT
tkv5 FRT
c
TkvACT Merge
Cell area x10² (μM²)
40
30
20
10
0
Control
TkvACT

Figure S1. The rapid removal of homozygous *Mad*$^{8-2}$ or *tkv*^{5} cells is delayed by chloroquine.

(**a**) Mosaic clones showing homozygous *Mad*$^{8-2}$ *(hsFLP; Mad*$^{8-2}$ *FRT40A/ Ubi-GFP FRT-40A)* or *tkv*^{5} *(hsFLP tkv*^{5} *FRT40A/ Ubi-GFP FRT 40A)* cells detected by the absence of GFP (green) have similar mCherry-Atg8a (red) puncta at -4 h RPF compared to the wild type cells yet, the *Mad*$^{8-2}$ or *tkv*^{5} are rapidly removed. Three representative images are shown. (**b**) The rapid removal of homozygous *Mad*$^{8-2}$ or *tkv*^{5} cells can be partially rescued by feeding the larvae chloroquine (CQ), compared to the vehicle (PBS), as *Mad*$^{8-2}$ or *tkv*^{5} cells from midguts from larvae fed CQ have intact nuclei and morphology. (**c**) Mosaic clone showing Tkv^{ACT} cells marked by GFP (green) have similar size compared to the neighboring control cells from earlier midguts (-8 h RPF). At this early stage autophagy levels are undetectable (pmCherry-Atg8a, red) with only background observed. Quantitation of cell size measured by ImageJ (average ± SD). (**a-c**) DNA is stained by Hoechst (blue). Scale bars represents 25 μm.

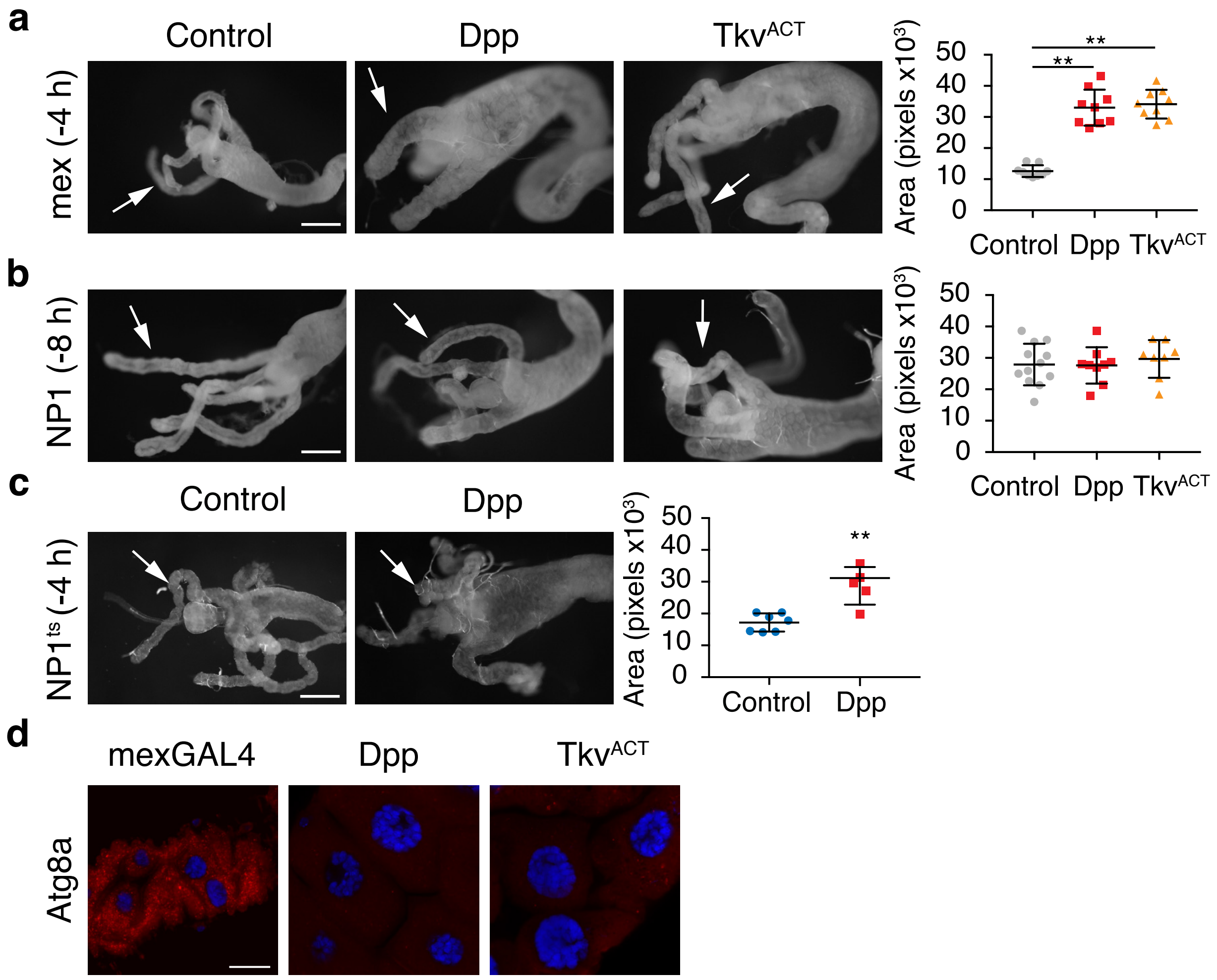

Figure S2. Sustained Dpp signaling prevents larval midgut degradation.

(**a**) Morphology of control *(mex-GAL4/+)*, Dpp *(mex-GAL4/+; UAS-dpp/+)* and Tkv^{ACT} *(mex-GAL4/+; UAS-tkv^{ACT}/+)* midguts from late third instar animals (-4 h RPF) shows enlarged midgut and gastric caeca (arrows). Quantitation of the gastric caeca size (average pixels ± SD) (**$p <$ 0.0001). (**b**) Morphology of control *(NP1-GAL4/+)*, Dpp *(NP1-GAL4/+; UAS-dpp/+)* and Tkv^{ACT} *(NP1-GAL4/+; UAS-tkv^{ACT}/+)* expressing midguts from early third instar animals prior to the onset of degradation (-16 to -8 h RPF) shows similar size. Quantitation of the gastric caeca size (average pixels ± SD). (**c**) Morphology of control *(NP1^{ts}/+)* and Dpp *(NP1^{ts}/+; UAS-dpp/+)* midguts at -4 h RPF from animals transferred to 29°C at early third instar shows a similar midgut phenotype to *NP1>dpp* and *mex>dpp*. Quantitation of the gastric caeca size (average pixels ± SD). (**$p =$ 0.0003). (**a-c**) Scale bar represents 200 µm. (**d**) Autophagy detected by Atg8a (red) staining in midgut cells from control, *mex>dpp* and *mex>tkv^{ACT}* larvae at -4 h RPF. DNA is stained by Hoechst (blue). Scale bar represents 20 µm.

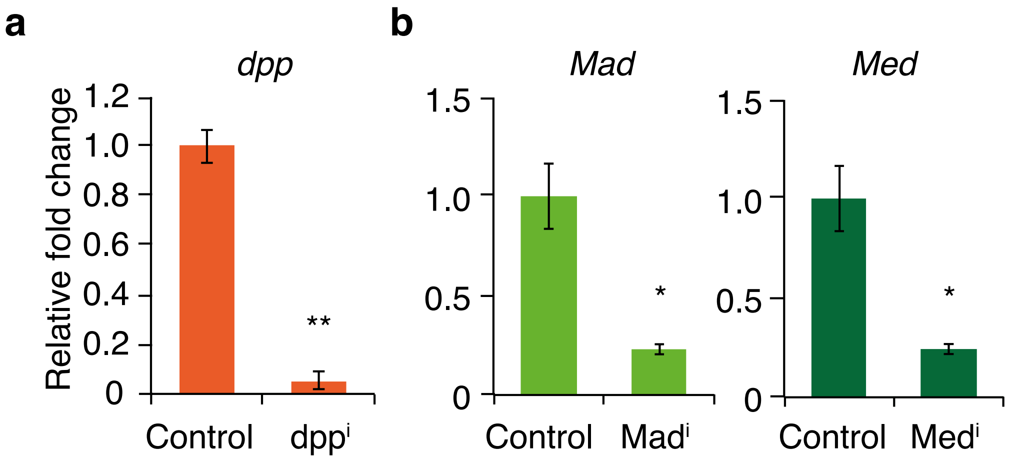

Figure S3. Quantitation of the level of RNAi knockdown in the midgut.

(**a**) Quantitation by qRT-PCR of the level of RNAi mediated knockdown of *dpp* (average ± SEM) (**p < 0.001). (**b**) Quantitation by qRT-PCR of the level of RNAi mediated knockdown of *Mad* and *Med* (average ± SEM) (*p < 0.05). Data are from 3 experiments, with 20 midguts per sample.

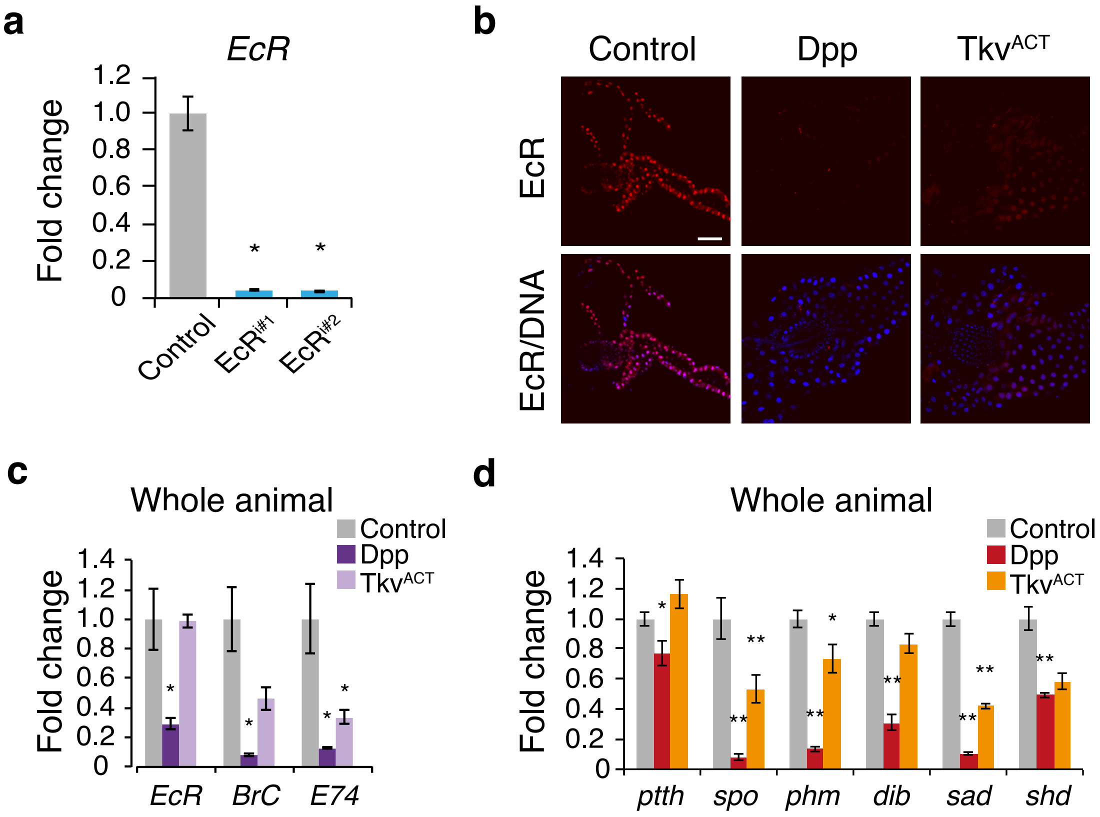

Figure S4. Dpp signaling impairs ecdysone production and signaling.

(**a**) Quantitation by qRT-PCR of the level of RNA*i* mediated knockdown of two independent *EcR* RNAi lines from -4 h RPF midguts (average ± SEM) (**p* < 0.001). Data are from 3 experiments, with 20 midguts per sample. (**b**) Control (*mex-GAL4/+*), *mex>dpp* and *mex>tkv*^{ACT} midguts from larvae at -4 h RPF stained with EcR antibody (red) show dramatically reduced EcR. DNA is stained by Hoechst (blue). Scale bar represents 100 µm. (**c**) The levels of *EcR, Br-C* and *E74* are reduced in *mex>dpp* and *mex>tkv*^{ACT} whole larvae. Transcript levels were measured by qRT-PCR at -4 h RPF. Data are from 3 experiments, with 3 animals per sample (average ± SEM) (**p* < 0.01). (**d**) Transcript levels of *ptth*, *spo, phm, dib, sad, shd* and *ecd* were measured by qRT-PCR from control, *mex>dpp* and *mex>tkv*^{ACT} late third instar larvae (-4 h RPF). Data are from 3 experiments, with 3 larvae per sample (average ± SEM) (***p* < 0.001).
